# Posture in Percussion of the Heart

**Published:** 1898-01-22

**Authors:** 


### POSTURE IN PERCUSSION OF THE HEART.

Observations on the "size" of the heart which have from time to time been made on patients undergoing treatment by baths and exercises have raised many questions, which cannot yet be regarded as definitely answered, in regard to the cause of the rapid alteration in the area of the cardiac dulness which has so often been observed. While some people have accepted unreservedly the idea that the heart may undergo rapid alteration in its size, and presumably in the capacity of its cavities, others have thought these observations must be taken as showing that the heart is a far more moveable organ, and that its relations with the chest wall are far more variable, than many have imagined to be the case.

Dr. Paterson, of Cardiff, has published in the *British Medical Journal* some very interesting observations on the effect of posture in percussion of the heart, which show how great is the influence of gravity on the position of the heart, and on the area of the chest wall with which it comes into contact. It would seem, in fact, that in consequence of our always examining the body after death in the supine position, we are apt to get very false ideas as to the position of the heart during life. By making sections of dogs killed and immediately frozen in dfferent postures, Dr. Paterson has shown that in the supine position a very large area of what in man would be the anterior surface of the heart comes against the chest wall, whereas when lying on the back the portion in contact is very small. When in the left side position, although the whole mass of the heart moved to the left, the amount of the surface in apposition was not increased.

These observations have led Dr. Paterson to examine patients by percussion while in the supine position. This may be done either by letting the patient lie on two tables, separated at the point where percussion is to be applied, or by merely making the patient lean forward so as to place the body in the horizontal position, supporting his weight by placing the right hand on some appropriate object. Under these circumstances a considerable change observed is in the area of absolute dulness, which is increased approximately to that usually occupied by the area of relative dulness, the only difference of moment being a somewhat wider limit for the upper and left boundary in the latter. With regard to the advantages of this method of examination, Dr. Paterson believes that it will be found to be much more precise than the estimation of the relative dulness in the supine position. A lighter stroke is required, the percussion note is more definite, the constant variation in the pulmonary borders is largely eliminated; and, having to deal with an increased area, giving an absolutely dull note, the accuracy of percussion is greater. Lastly, it brings out an area of dulness which it may be quite impossible to ascertain in the supine position. Clearly the method is useful in many ways. Unfortunately, a new clinical experience must be gathered together before the meaning of its indications can be fully appraised, and we must always remember that, while cardiac dulness remains the complement of pulmonary resonance, it must always partake of the errors to which any single sign is liable.

